# Reconfiguration of the plastid genome in *Lamprocapnos spectabilis*: IR boundary shifting, inversion, and intraspecific variation

**DOI:** 10.1038/s41598-018-31938-w

**Published:** 2018-09-11

**Authors:** Seongjun Park, Boram An, SeonJoo Park

**Affiliations:** 1 Institute of Natural Science, Yeungnam University, Gyeongsan, Gyeongbuk 38541 South Korea; 2 Department of Life Sciences, Yeungnam University, Gyeongsan, Gyeongbuk 38541 South Korea

## Abstract

We generated a complete plastid genome (plastome) sequence for *Lamprocapnos spectabilis*, providing the first complete plastome from the subfamily Fumarioideae (Papaveraceae). The *Lamprocapnos* plastome shows large differences in size, structure, gene content, and substitution rates compared with two sequenced Papaveraceae plastomes. We propose a model that explains the major rearrangements observed, involving at least six inverted repeat (IR) boundary shifts and five inversions, generating a number of gene duplications and relocations, as well as a two-fold expansion of the IR and miniaturized small single-copy region. A reduction in the substitution rates for genes transferred from the single-copy regions to the IR was observed. Accelerated substitution rates of plastid *accD* and *clpP* were detected in the *Lamprocapnos* plastome. The accelerated substitution rate for the *accD* gene was correlated with a large insertion of amino acid repeat (AAR) motifs in the middle region, but the forces driving the higher substitution rate of the *clpP* gene are unclear. We found a variable number of AARs in *Lamprocapnos accD* and *ycf1* genes within individuals, and the repeats were associated with coiled-coil regions. In addition, comparative analysis of three Papaveraceae plastomes revealed loss of *rps15* in *Papaver*, and functional replacement to the nucleus was identified.

## Introduction

Angiosperm plastid genomes (plastomes) generally exhibit two copies of an inverted repeat (IR) region, referred to as IR_A_ and IR_B_^1^. The two IR copies are separated by two single-copy (SC) regions: the large single copy (LSC) and the small single copy (SSC). Plastome architecture and gene synteny are generally conserved^1,2^, but comparative analyses have revealed lineage-specific rearrangements within angiosperm plastomes. Notable examples of plastome rearrangements are found in Campanulaceae^3,4^, Fabaceae^5^, Geraniaceae^6,7^, and Oleaceae^8^. Homologous recombination between repeated sequences causes inversions in plastomes, which is considered the main mechanism underlying changes in gene order^2^. tRNA genes or some combination of repeats and tRNA may also facilitate genome rearrangements, including inversions^4,9,10^. The distribution, proportion, and number of repeats are correlated with the degree of genome rearrangement^11,12^.

Some of the structural rearrangements within plastomes involve IR boundary shifts (expansions and contractions), which have contributed to extensive rearrangements. For example, the sizes of IRs are extremely variable due to IR boundary shifts, ranging from 7 kb in *Monsonia speciosa*^7^ to 88 kb in *Pelargonium transvaalense*^13^, though the angiosperm IR is typically 25 kb. IR expansions and contractions also cause variation in gene content because of the transfer of genes from SC regions into the IR or otherwise. Gene conversion, double-strand breaks (DBS), and genomic deletion have been proposed as possible mechanisms of IR boundary shifts^14–17^. Although the presence of the IR may play an important role in plastome stability among photosynthetic angiosperms, loss of the IR has been observed in the inverted repeat-lacking clade of papilionoid legumes^18^, saguaro cactus^19^, and some species of *Erodium*^7,20^.

The angiosperm family Papaveraceae (poppy family) *sensu lato* (*s*.*l*.) comprises approximately 775 species in 42 genera distributed throughout the world^21^. The poppy family includes economically and medically important plants that produce the various pharmaceutical resources, particularly in the form of alkaloids^22^. The poppy family exhibits extensive morphological diversity, especially in its floral organ^23,24^. Thus, Papaveraceae is an ideal family for addressing fundamental questions about the genetic architecture of flowers and floral diversification. A robust phylogenetic reconstruction of this family is required to address these questions. However, previous phylogenetic analyses left some of the deepest nodes in the subfamily unresolved or weakly supported. For example, based on molecular (*rbcL*, *matK*, *trnL-F*, and 26 S nuclear ribosomal DNA) and morphological data^25^, Papaveraceae *s.l*. has been classified into two subfamilies: Fumarioideae (DC) Endl. (including *Pteridophyllum* and *Hypecoum*) and Papaveroideae Eaton. Recent research based on three plastid loci (*atpB*, *rbcL*, and *matK*) and 26 S nuclear ribosomal DNA showed that *Pteridophyllum* was an early-diverging genus in Papaveraceae^26^. Complete plastomes could present challenges regarding the evaluation of patterns of molecular evolution and provide strong support for deep phylogenomic relationships. However, complete plastome sequences have been reported for only two species of Papaveraceae^27,28^, neither of which is a member of the subfamily Fumarioideae.

As part of our ongoing research on the evolution of plastome and phylogenomic relationships among Papaveraceae, we uncovered the plastome sequence of *Lamprocapnos spectabilis*, representing the first sequenced member of the subfamily Fumarioideae. *Lamprocapnos* Endlicher is a monotypic genus in which *L*. *spectabilis* is the only species. This species is an economically and horticulturally important endemic plant native to Korea, Japan, northern China, Siberia. Comparison of three plastomes (from *Lamprocapnos*, *Papaver* and *Coreanomecon*) revealed extensive diversity in terms of size, structure, gene content, and substitution rates in *Lamprocapnos*. We tested for an effect of the IR on the substitution rates of genes transferred from the SC regions into the IR. We found intraspecific length variation in the *accD* and *ycf1* coding regions of the *Lamprocapnos* plastome. In addition, we identified loss of the plastid *rps15* gene in *Papaver* and putative functional transfer of this gene to the nucleus. Our results provide new insights into the evolution of plastomes within the family Papaveraceae.

## Results

### Organization of the *Lamprocapnos* plastome

**Figure 1:**
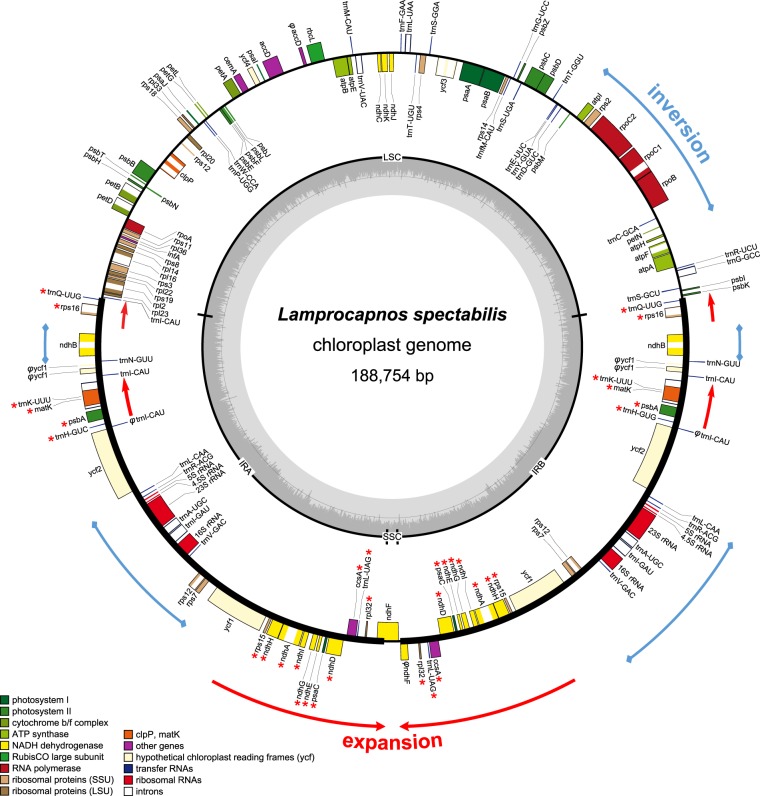
Circular gene map of the *Lamprocapnos spectabilis* plastome. Thick lines on the inner circle indicate the inverted repeats (IR_A_ and IR_B_, 51,309 bp), which separate the genome into small (SSC, 1,645 bp) and large (LSC, 86,358) single-copy regions. Genes on the inside and outside of the map are transcribed in clockwise and counterclockwise directions, respectively. The ring of bar graphs on the inner circle indicates the GC content in dark grey. Asterisks indicate genes transferred from single-copy regions to the IR and φ denotes a pseudogene.

**Figure 2:**
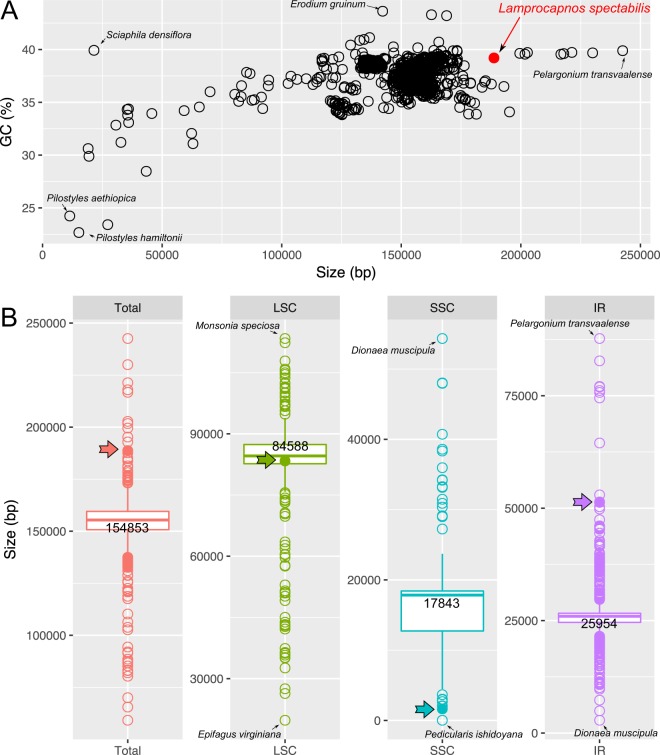
All complete angiosperm plastomes from the NCBI Genome database, accessed on January 1, 2018. (**A**) Size and GC content (red) of the *Lamprocapnos spectabilis* plastome relative to 1,936 angiosperm plastomes from the NCBI Genome database. (**B**) Boxplot distribution of the sizes of the total genomes (red), large single-copy (LSC, green), small single-copy (SSC, blue) and inverted repeat (IR, purple) among 1,857 angiosperms containing two IRs. The numbers on the boxes indicate the median genome size, LSC, SSC and IR. Arrows with a closed circle indicate the positions of *L*. *spectabilis*.

The assembled complete plastome of *L. spectabilis* is 188,754 bp in length, with an average mean coverage depth of 5,001-fold (Fig. S1). It exhibits a typical quadripartite architecture, with a pair of IRs of 51,384 bp separated by SSC and LSC regions of 1,645 and 83,341 bp, respectively (Fig. 1 and Table S1). The plastome of *L. spectabilis* is larger than the median genome size (154,853 bp) for the 1,936 sequenced angiosperm plastomes (National Center for Biotechnology Information; NCBI, accessed on January 1, 2018, Fig. 2). The GC content of the *Lamprocapnos* plastome (39.2%) is higher than the median GC content (37.6%) of the selected angiosperm plastomes (Fig. 2A). The size of the LSC in the *Lamprocapnos* plastome is close to the median genome size (84,588 bp) for the selected angiosperm plastomes; however, the 1,645 bp SSC is the smallest SSC found in any plastome analyzed to date (except for the hemiparasite *Pedicularis ishidoyana*, Fig. 2B). The IR is greatly expanded at the IR_B_/LSC, IR_B_/SSC and IR_A_/SSC boundaries relative to the model plant tobacco (*Nicotiana tabacum*, NC_001879) and was two times longer than the median size (25,954 bp) for the selected angiosperm plastomes (Fig. 2B). Expansion at the IR_B_/LSC boundary has resulted in the duplication of six genes, from *trnQ-UUG* to *trnH* (Fig. 1). The IR_B_/SSC expansion includes 12 genes, from *ycf1* to *rpl32*, and the IR_A_/SSC expansion includes an N-terminal portion of *ndhF* (753 bp) generating a truncated *ndhF* fragment in IR_B_ (Fig. 1). These expansions result in a very small SSC containing the C-terminal portion of *ndhF* (Fig. 1). The plastome contains 79 unique protein genes (18 of which are duplicated in the IR), 30 tRNA genes (11 are duplicated in the IR, including two *trnI*-CAU, for which a fifth copy is located in the LSC), and four rRNA genes (all of which are duplicated in the IR), with 21 introns (20 *cis*-spliced and 1 *trans*-spliced) (Table S1). The *Lamprocapnos* plastome exhibits a higher percentage of dispersed and tandem repeats than the two other plastomes of Papaveraceae, *Papaver* and *Coreanomecon*, respectively (Table S1). Both types of repeats in the *Lamprocapnos* plastome are presented in Fig. S1.

The original copy of *trnI-CAU* upstream from *ycf2* is predicted by tRNAscan-SE to be a pseudogene (Fig. S2A). Alignment of the three copies (two copies excluded because they were duplicated in the IR) of *Lamprocapnos trnI-CAU* to *Euptelea* shows pairwise identities ranging from 93.2% to 94.6%, whereas *Papaver* and *Coreanomecon* exhibit sequences identical to the *Euptelea trnI*-*CAU* copy (Fig. S2B). The original copy of *trnI-CAU* contains five base substitutions, compared with the three related species (Fig. S2B). Three point mutations (nucleotide 24 of the D-stem and nucleotides 52 and 53 of the T-stem) result in destabilization of base-pairing in both the D- and T-stems (Fig. S2C). The other two duplicated copies present four base substitutions, but without impairment of the secondary structure (Fig. S2B and S2C). The base substitutions in three *trnI-CAU* copies were confirmed by Sanger sequencing.

### Structural evolution of the *Lamprocapnos* plastome

**Figure 3:**
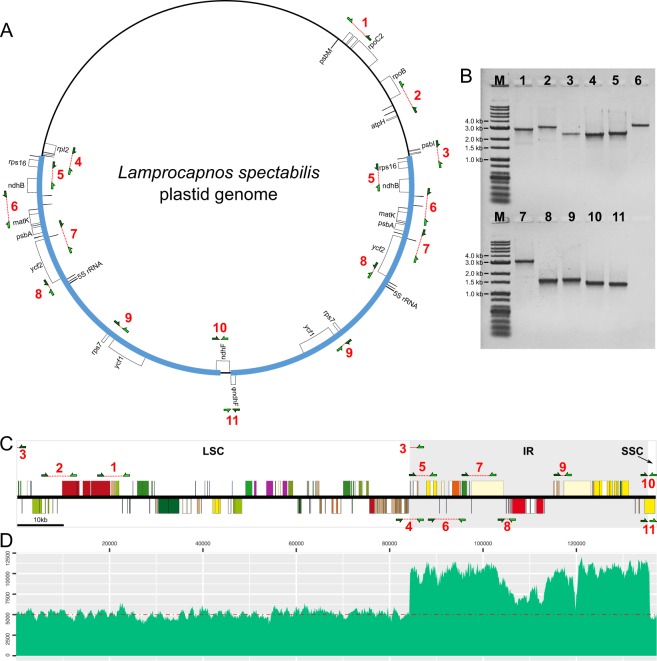
Analysis of plastome arrangements in *Lamprocapnos*. (**A**) PCR strategy for detecting inversions and translocations. Arrowheads with red lines on the inside or outside of the circle indicate the position and orientation of the PCR primers used to confirm the structure of the plastid genome. (**B**) Assay results using primers designed to amplify 11 regions. Lane M contains the SolGent^TM^ 1 kb plus DNA ladder, and lanes (1–11) correspond to the numbers of the 11 PCR amplicons in (**A**) or (**C**). The full-length electrophoretic gel is presented in Supplementary Fig. 12. (**C**) Linear plastome map of *Lamprocapnos* with the 11 PCR amplicon sets. The genes above and below the horizontal line correspond to the genes in Fig. 1. (**D**) Graph showing the base per base depth of the sequencing coverage across the *Lamprocapnos* plastome with one IR region. The red dot-dashed line indicates the coverage of the plastome with two IRs.

**Figure 4:**
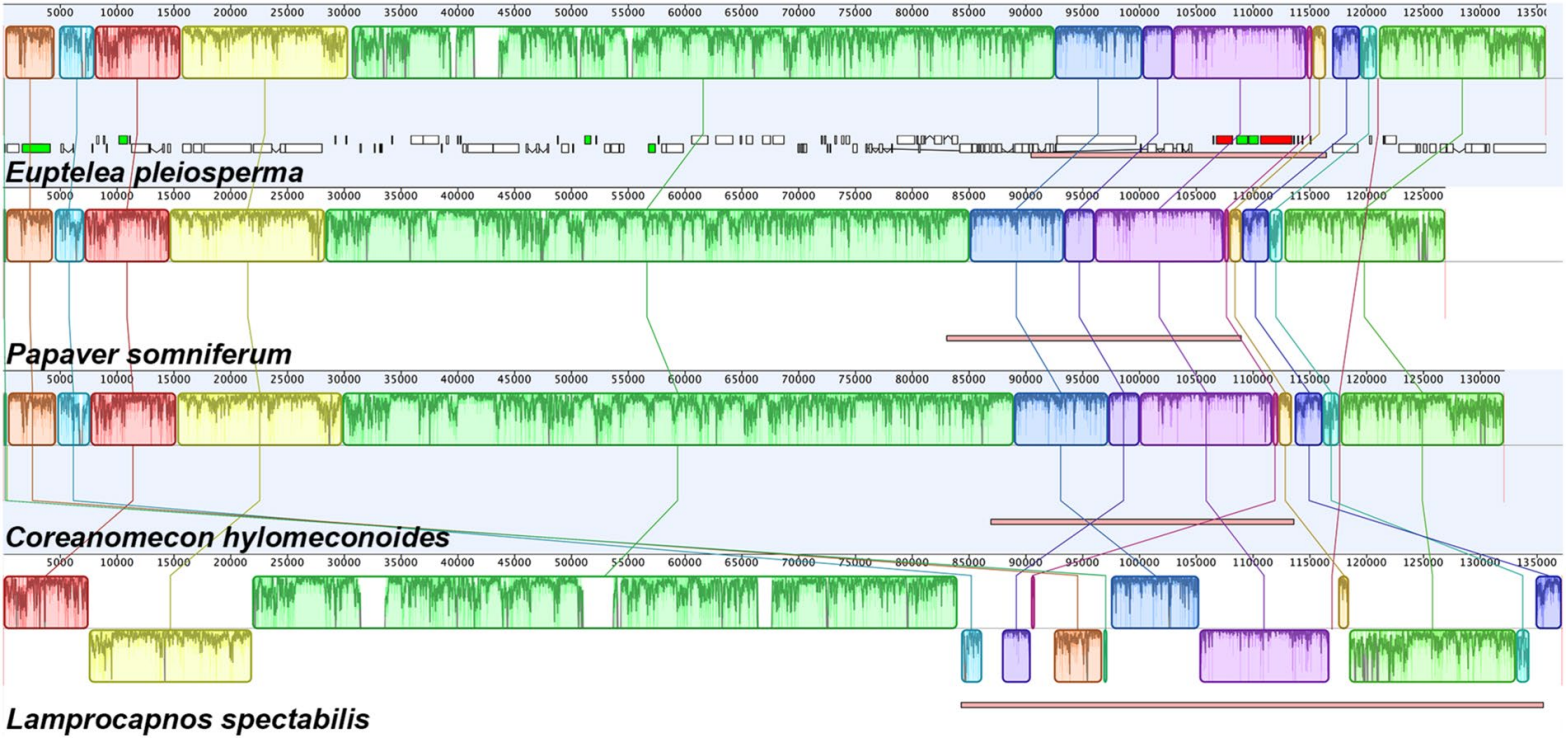
Structural alignments of Papaveraceae plastomes using Mauve. The colored blocks represent collinear sequence blocks shared by all plastomes. Blocks drawn below the horizontal line indicate sequences found in an inverted orientation. Individual genes and strandedness are represented below the *Euptelea* genome block. Only one copy of the inverted repeat (IR) is shown for each plastome and pink boxes below each plastome block indicate its IR.

**Figure 5:**
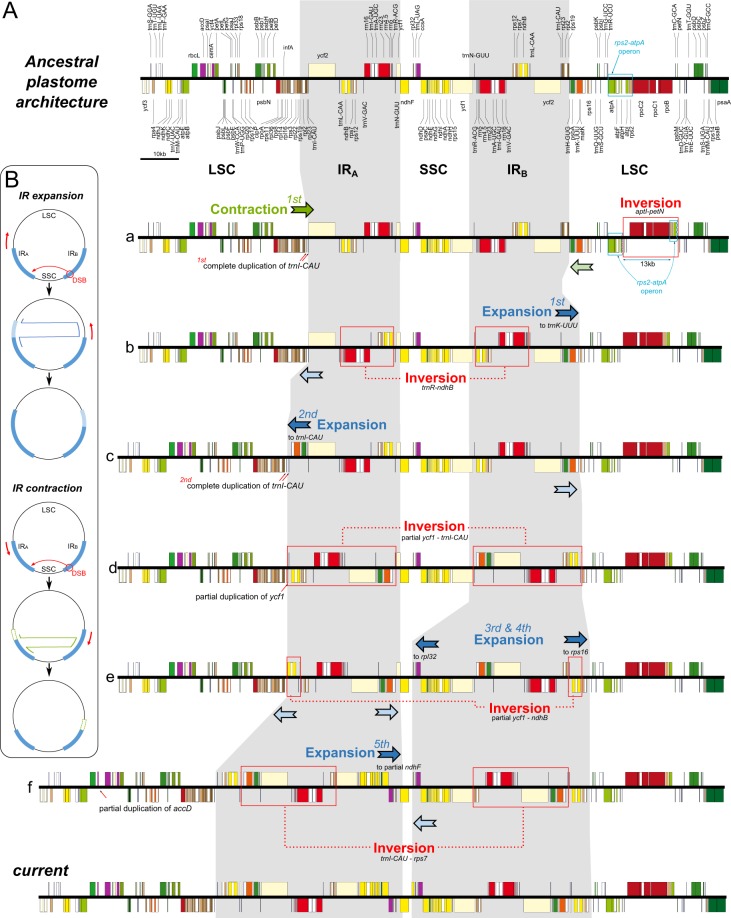
Model of plastome rearrangement in *Lamprocapnos*. (**A**) The ancestral plastome architecture of Papaveraceae (top), the hypothetical intermediate states (a to f), and the current *Lamprocapnos* plastome (bottom) are shown. Ancestral angiosperm genome structure is represented by *Nicotiana tabacum*, which is also conserved with gene order identical to three related species (*Papaver*, *Coreanomecon*, and *Euptelea*). The genes above and below the horizontal line are transcribed in rightward and leftward directions, respectively. Gray shading highlights inverted repeat (IR) regions with IR boundary shifts. The colored arrows correspond to the IR boundary shifts (dark green, the first IR contraction; light green, the subsequent IR contraction; dark blue, the first IR expansion; light blue, the subsequent IR expansion). The red boxes indicate the inferred inversion regions. The blue box indicates the *rps2-atpA* operon regions. (**B**) The hypothetical model for IR expansion and contraction is illustrated. IR expansion is generated with a double-strand break (DBS) event in IR_B_, followed by strand invasion, expansion, and recombination in IR_A_. IR contraction can likely occur via a similar mechanism. IR expansion and contraction can also occur from different directions.

The *Lamprocapnos* plastome exhibits increased levels of structural divergence in comparison with *Nicotiana tabacum*. To confirm the structural divergence of the *Lamprocapnos* plastome, we designed 11 sets of polymerase chain reaction (PCR) primers that specifically targeted the predicted rearrangement boundaries by amplifying across junctions (Fig. 3A,C). The obtained PCR products verified the existence of structural divergence in the plastome (Fig. 3B). Twice the sequencing depth being obtained in the IR region provides clear evidence of the expanded IR and miniaturized SSC (Fig. 3C,D). In comparison with related species from three genera (*Papaver*, *Coreanomecon*, and *Euptelea*), *L. spectabilis* has experienced numerous changes in its plastome structure (Fig. 4). Mauve alignment led to the identification of 14 locally collinear blocks (LCBs) shared by two Papaveraceae species and the outgroup (Fig. 4). The LCBs identified in the *L. spectabilis* plastome suggest that it has experienced eight inversions involving 14 breakpoints. The breakpoints were inferred to have occurred at *trnQ-psbK*, *atpH-petN*, *atpI-psbM*, *trnI-trnQ*, *rps16-ndhB*, *ndhB-trnN*, *trnN-trnK*, *psbA-trnH*, *trnH-trnI*, *trnL-trnR*, *trnV-rps12*, *rps7-ycf1*, *trnL-rpl32*, and *rpl32-ndhF*. At least some of the inversions are likely the result of expansions or contractions of IRs. The *Lamprocapnos* plastome was compared with the *Nicotiana tabacum* plastome as an ancestral plastome architecture to identify genome rearrangement events (Fig. 5). A plastome rearrangement model that explains IR boundary shifts, inversions, operon disruption, and gene duplications and relocations was proposed for *Lamprocapnos* (Fig. 5). Many of the inversions in the *Lamprocapnos* plastome occurred within the IRs, whereas only one inversion, causing disruption of the *rps2-atpA* operon, was located in the LSC region (Fig. 5). Numerous genes and gene fragments are duplicated, including sequences from *ccsA*, *matK*, *ndhA*, *ndhD*, n*dhE*, *ndhF*, *ndhG*, *ndhH*, *ndhI*, *psaC*, *psbA*, *rpl32*, *rps15*, *rps16*, *trnH-GUG*, *trnI-CAU*, *trnK-UUU*, *trnL-UAG*, and *trnQ-UUG*. Most of the duplications involve genomic rearrangements; however the partial duplication of *accD* is not associated with any inferred inversion or and IR boundary shift.

### Divergence of plastid-encoded *accD* and *ycf1* genes in the *Lamprocapno*s plastome

**Figure 6:**
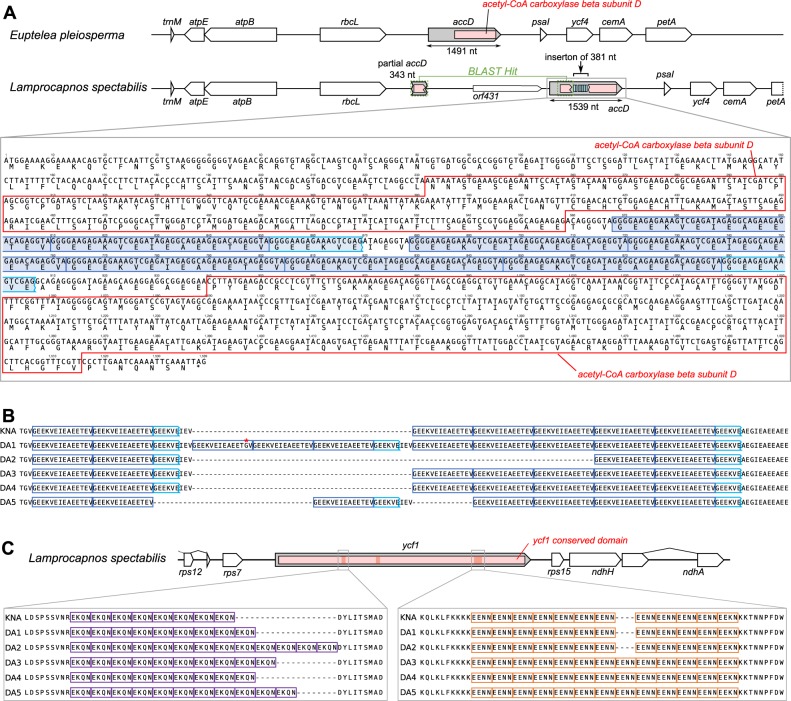
Length variation in plastid *accD* and *ycf1* of *Lamprocapnos*. (**A**) Schematic diagram of the genomic regions surrounding plastid *accD*. Boxes inside the *accD* gene (gray) indicate the conserved domain (acetyl-CoA carboxylase beta subunit; pink). The nucleotide and amino acid sequences of the plastid *Lamprocapnos accD* gene are shown in detail. Red boxes indicate the conserved domain of the acetyl-CoA carboxylase beta subunit. Blue boxes indicate amino acid repeat (AAR) motifs. (**B**) Amino acid sequences of plastid *accD* copies from six *Lamprocapnos* individuals. The dark and light blue boxes correspond to each AAR motif in (**A**). An asterisk indicates an amino acid sequence mismatch of the AAR motif. (**C**) Schematic diagram of genomic regions surrounding the plastid *ycf1*. Boxes inside the *ycf1* gene (gray) indicate the conserved domain (pink) and three hotspot regions (red). Each amino acid sequence of the two hotspot regions of *ycf1* copies from six *Lamprocapnos* individuals. Purple and orange boxes indicate amino acid repeat (AAR) motifs.

The *Lamprocapno*s plastome contains one partial and one complete copy of the acetyl-CoA carboxylase β subunit (*accD*) in the LSC region (Fig. 6A). The partial *accD* copy (343 bp) is located downstream of the *rbcL* gene, which is similar to the N-terminal portion of the complete copy, with 80.5% identity (Figs 6A and S3). Compared with three related species (*Papaver*, *Coreanomecon*, and *Euptelea*), the *Lamprocapno*s *accD* gene exhibits an insertion of amino acid repeats (AARs) resulting in the conserved domain being split into two portions (Fig. S4). The AARs of the *Lamprocapno*s *accD* gene contained seven repeats of the “GEEKVEIEAEETEV” motif and two partial repeats of “GEEKVE” (Fig. 6A). Our RT-PCR results showed that the plastid-encoded *accD* gene was actively transcribed (Fig. S5A). The insertions were confirmed by sequencing of both the PCR and RT-PCR products. A coiled-coil region (CCR) was predicted in some of the AARs of the *Lamprocapno*s *accD* gene but not in those of the other related species (Fig. S6). Because we were intrigued by the 1,296 bp of the open reading frame (ORF) (*orf431*) upstream from the complete *accD* copy (Fig. 6A), we next decided to perform RT-PCR analysis. The results indicated that *orf431* was also transcribed but was not co-transcribed with the *accD* gene (Fig. S5A). *orf431* is strongly predicted to encode a protein with a transmembrane domain (Fig. S5B), but *orf431* could not be identified in a blastn search against the NCBI non-redundant (nr) nucleotide database. Similarly, we identified AAR motifs in the *Lamprocapno*s *ycf1* gene (Fig. S7). Amino acid alignment of four *ycf1* copies from *Papaver*, *Coreanomecon*, *Lamprocapno*s, and *Euptelea* showed that the *Lamprocapno*s *ycf1* copy harbors three insertions containing AAR motifs: 1) eight repeats of “EKQN”, 2) six repeats of “EAQERE”, and 3) 12 repeats of “EENN” (Fig. S7). Small CCRs were also detected in all four *ycf1* copies (Fig. S8), but only the small CCRs of *Lamprocapno*s include AAR motifs.

To gain further insight into the variability of the AAR motifs in the plastid *accD* and *ycf1* coding regions at an individual level, we sequenced the hotspot regions including the AARs from an additional five individuals of *L. spectabilis* (Table S2). Alignment of the inserted region sequences of the six individuals revealed intraspecific variation of the *accD* and *ycf1* coding regions in the *Lamprocapnos* plastome (Fig. 6B,C). The length of the *accD* coding region ranges from 91 to 184 amino acids, including 1) four to 10 repeats of “GEEKVEIEAEETEV” and 2) two or three repeats of “GEEKVE” (Fig. 6B). In the case of the *ycf1* gene, two hotspot regions show length variation, consisting of 1) 8 to 13 repeats of “EKQN” and 2) 12 or 13 repeats of “EENN (Fig. 6C).

### Nucleotide substitution rates

**Figure 7:**
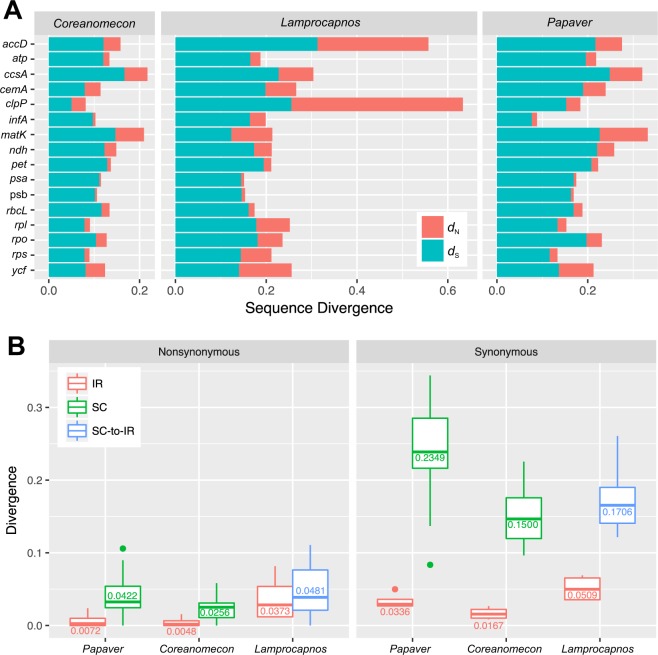
Variation in sequence divergence among *Lamprocapnos*. (**A**) Sequence divergence among *Coreanomecon*, *Lamprocapnos*, and *Papaver* plastid genes or functional groups of genes. (**B**) Boxplot distribution of the relative rates of *d*_N_ and *d*_S_ values for SC-to-IR genes from *Lamprocapnos*.

The examination of the nonsynonymous and synonymous divergence of individual genes revealed heterogeneity of the rate of nonsynonymous substitutions in *Lamprocapno*s plastid genes (Fig. 7). Acceleration of the rate of nonsynonymous substitutions was observed for the *accD* and *clpP* genes in *Lamprocapno*s (Fig. 7A). Four genes, *clpP*, *rpl23*, *rpl36*, and *ycf2*, showed *d*_N_/*d*_S_ ratios greater than one (Fig. S9), while only three genes (*clpP*, *rpl36*, and *ycf2*) exhibited a significantly different *d*_N_/*d*_S_ in the likelihood ratio tests (LRTs) (*p* < 0.05 after Bonferroni’s correction; Table S3). Mapping the nonsynonymous and synonymous rates onto the genome showed a low correlation between the rearrangements and nucleotide substitution rates (Fig. S10).

To examine the effect of the IR on plastome substitution rates, we used 17 protein-coding genes (Fig. 1) shared by three species and compared the *d*_N_ and *d*_S_ values of the genes in the IR and SC regions (Fig. 7B). The genes in the *Papaver* and *Coreanomecon* SC regions present a higher *d*_N_ and *d*_S_ than those in the IR regions (*Papaver*, *d*_N_: 5.9-fold, *d*_S_: 7.0-fold; *Coreanomecon*, *d*_N_: 5.3-fold, *d*_S_: 9.0-fold). However, the *d*_N_ and *d*_S_ of SC-to-IR-shifted genes in *Lamprocapno*s are 1.3-fold and 3.5-fold higher, respectively, than those of the genes in the IR.

### Characterization of putative functional gene transfer to the nucleus

Compared with three related species (Fig. S11A), the plastid-encoded ribosomal protein S15 (*rps15*) is truncated in *Papaver*, leaving only 75 bp at the N-terminus. Three internal stop codons have been generated through insertions/deletions and substitutions, which lack the most conserved domain, suggesting that *Papaver rps15* is a pseudogene (Fig. S11A). Transfer of the *rps15* gene from the plastid to the nucleus was detected in the *Papaver* transcriptome data (Fig. S11B). The first 78 amino acids of a predicted ORF are strongly predicted by TargetP to constitute a chloroplast transit peptide (cTP = 0.964) (Fig. S11B).

## Discussion

In this study, we first generated the complete plastome of *Lamprocapno*s *spectabilis* from the subfamily Fumarioideae (Papaveraceae). The *Lamprocapno*s plastome has experienced multiple genomic changes, including inversions, operon disruption, gene relocation and duplication, and IR shifts that distinguished it from the two other sequenced Papaveraceae. We propose a rearrangement model for the *Lamprocapno*s plastome to explain a complicated structure (Fig. 5). Two factors, IR boundary shifts and inversions, are considered the main mechanisms responsible for the genomic changes observed in the *Lamprocapnos* plastome. The IR expansions generated dramatic IR and SSC size changes in the *Lamprocapnos* plastome, resulting in an IR two times longer than the median size and the smallest SSC among the analyzed angiosperms. A possible mechanism leading to the larger IR expansion in the *Lamprocapnos* plastome may involve a DSB followed by strand invasion, expansion, and recombination in the IR^14^. Extreme expansions (exceeding 20 kb) have been reported for a few angiosperm lineages, such as *Annona*^29^, *Erodium*^20^, and *Pelargonium*^13^. Among angiosperms, most of the inversions found plastomes are located in the LSC region^8,30–33^, while most inversions in the *Lamprocapnos* plastome occurred within the IRs. Similar inversions that occur in the IRs were observed in two *Plantago* plastomes^34^. The distribution of dispersed and tandem repeat sequences in the *Lamprocapnos* plastome (Fig. S1) suggested that the inversions may be associated with repeats.

IR boundary shifts and inversions can be sources of gene duplication. The position of *trnI-CAU* in the *L. spectabilis* plastome next to the IR boundary suggested that duplications of *trnI-CAU* may result from a series of IR contractions and expansions. Otherwise, partial duplications of the *ycf1* gene are likely due to inversion, but some combination of IR boundary shifts and inversions may be possible. In addition to *trnI-CAU* and *ycf1*, 13 protein-coding genes are completely duplicated in the IR, which transferred from LCS or SSC genes to the IR. IR genes generally exhibit lower synonymous substitution rates than SC genes^35,36^. Furthermore, relocated genes transferred from SC regions to the IR tend to decrease substitution rates via copy-dependent repair activity^34^. Our comparisons also revealed a reduction of the substitution rate for SC-to-IR genes in the *Lamprocapnos* plastome (Fig. 7).

The angiosperm plastomes encode three tRNAs with a CAU anticodon: tRNA^fMet^ (*trnfM-CAU*), tRNA^Met^ (*trnM-CAU*) and tRNA^Ile^ (*trnI-CAU*), all three of which are essential^37^. The AUA codon is read by tRNA^Ile^ (*trnI-CAU*) in plastids, in which the cytidine in the wobble position of the anticodon is converted by a post-transcriptional modification^38^. Due to the effect of genomic rearrangements, the *Lamprocapnos* plastome contains five copies of *trnI-CAU* (four copies located in IRs and one copy in the LSC) and the original copy upstream from *ycf2* have base-pairing issues in two D- and T-stems of *trnI-CAU* (Fig. S2). Among these two stems, the D-stem plays a more important role in the tertiary structure and folding of tRNA^39^. Moreover, the D-stem is associated with a recognition site for the specific enzyme aminoacyl-tRNA synthetase, which activates the amino acid^40–42^. Thus, the base-pairing issues observed in the original copy may influence aminoacylation due to the specific interaction of their loops in the tRNA tertiary structure. Alternatively, the other copies could act as a physical link between the coding information (the AUA codon) and the amino acid for isoleucine. Among angiosperm plastomes, duplication of *trnI-CAU* is observed in some species of *Paris*^43,44^ and *Trillius*^45^ from Melanthiaceae. However, the duplications in these lineages are likely due to only simple tandem duplication, and the duplicated copies of *trnI-CAU* are identical. In contrast, we assume that the duplications of *trnI-CAU* found in the *Lamprocapnos* plastome resulted from the combination of an inversion and IR shifts (tandem duplication).

A locus-specific rate increase compared with two Papaveraceae plastomes was observed in *Lamprocapnos* (Figs 7 and S9). In particular, the plastid-encoded *clpP* gene exhibits accelerated nonsynonymous substitution rates, with *d*_N_/*d*_S_ ratios greater than one (Fig. S9). The LRT suggested that the *clpP* gene has been under positive or relaxed purifying selection. Similar positive selection on the *clpP* has been observed in *Geranium*^46^, legume^47^, and *Silene*^48^ species, and the elevated substitution rates of the *clpP* genes in three lineages are correlated with the loss of introns. However, *Lamprocapnos clpP* presents a typical structure with two introns, arguing against mutagenic retroprocessing.

Interestingly, we discovered that the plastid-encoded *accD* harbors AAR motifs and that this gene exhibits elevated nonsynonymous and synonymous substitution rates (Figs 6 and 7). The large insertion of AAR motifs in the middle region may have caused the accelerated substitution rate of the *Lamprocapnos accD* gene. Previous studies have shown a correlation between indels (insertions and deletions) and nucleotide substitutions^49^, especially for repeated hydrophilic residues^50^, because they may act as mutagenic drivers^51^. Interruption of the plastid-encoded *accD* was recently identified in *Geranium*, showing a positive effect of the insertion on the nucleotide substitution rate of the *accD* gene^46^. The insertion of AAR motifs can influence the function of *accD*, but the absence of a frameshift mutation and the RT-PCR results suggested functionality of this gene. Thus, the split domains of *Lamprocapnos accD* may be linked by a coiled-coil, as alpha-helical CCR could mediate interactions between the two domains^52^. However, the insertions can modify protein interaction interfaces, causing a loss of protein-protein interactions^53^. Plastid-encoded *accD* is one component of the acetyl-CoA carboxylase (ACC) complex^54^. Thus, the large insertions of AAR motifs in the *Lamprocapnos accD* gene may influence the coordination between plastid-encoded *accD* and nuclear-encoded proteins (i.e., the acetyl-CoA carboxylase subunit α, *accA*; the biotin carboxyl carrier protein subunit, *accB*; and the biotin carboxylase subunit, *accC*). If the AAR motifs are deleterious mutations, they may precede potential functional replacement of the *Lamprocapnos accD* gene via gene transfer to the nucleus or gene substitution of eukaryotic ACCases. This scenario is supported by a recent study examining the evolutionary fate of the plastid-encoded *accD* gene in Geraniaceae^46^. For full understanding of the evolutionary fate of the plastid-encoded *accD* among *Lamprocapnos* genomes, complete sequences of the nuclear transcriptome will be required. Moreover, the AAR motifs may contribute to the phenotypic differences between *Lamprocapnos* individuals. Intraspecific length variation in the *accD* and *ycf1* gene sequences was identified in *Lamprocapnos* (Fig. 6). Length polymorphism due to indels in the genomic region is likely to be generated through replication slippage and recombination^55^. Insertion of repetitive amino acid sequence regions has been found in the *Medicago accD* and *ycf1* gene, and recombination activity in the repeats within these genes has been suggested^56^.

Angiosperm plastomes have retained remnants of 21 ribosomal protein subunits following endosymbiotic gene transfer^57^. Loss of eight genes (e.g., *rpl20*, *rpl22*, *rpl23*, *rpl32*, *rps7*, *rps11*, *rps16*, and *rps18*) has been reported in photosynthetic angiosperms^58^. However, successful functional replacement of the ribosomal protein subunits via gene transfer or substitution in angiosperms has been documented for only two gene transfer events (*rpl22* in Fabaceae^59^ and Fagaceae^60^ and *rpl32* in Ranunculaceae^61^, Rhizophoraceae^62^, and Salicaceae^63^) and two gene substitution events (*rps1*6 in *Medicago*^64,65^ and *Populus*^64^ and *rpl23* in *Geranium*^66^ and *Spinacia*^67^) because nuclear-encoded genes for plastid-targeted proteins must acquire a transit peptide that is transported from the cytoplasm into the plastids^68,69^. We identified loss of plastid-encoded *rps15* in the *Papaver* plastome, which was previously annotated as a functional gene^27^. Our analysis showed that the *rps15* gene is truncated and lacks the N-terminal conserved domain (Fig. S11A). We identified a nuclear transcript that contains a predicted plastid transit peptide with an intact conserved domain (Fig. S11B), suggesting successful functional replacement of the *rps15* in the nucleus via intracellular gene transfer (IGT). This is the first report of loss/transfer of the plastid-encoded *rps15* gene among angiosperms.

## Materials and Methods

### Genome sequencing, assembly and annotation

Fresh leaf tissue was obtained from a single individual of *Lamprocapnos spectabilis* at the Korea National Arboretum (KNA), Pocheon-si, South Korea. Total genomic DNA was isolated from 200 mg of *L. spectabilis* leaf tissue using the methods of Allen *et al*.^70^. The *Lamprocapnos* DNA (4.2 μg) was sequenced using the Illumina HiSeq. 2000 platform (Illumina, San Diego, CA) at LabGenomics (Seongnam, South Korea), generating 71.2 million 100 bp of paired-end (PE) reads from a 550 bp library.

The genome sequence data were assembled *de novo* with Velvet v1.2.10^71^ using multiple *k*-mers on a 12-core 3.33 GHz Linux work station with 192 GB of memory. From each assembly, the largest contig representing a complete plastome with only one copy of the IR was generated. To determine the whole plastome sequences, the initial plastid contigs were aligned and manually checked in Geneious R7 v7.1.8 (www.geneious.com)^72^. To assess the depth of coverage for the completed genome, Illumina PE reads were mapped to the whole plastome sequence with Bowtie v2.2.9^73^. The plastome was annotated using Dual Organellar GenoMe Annotator (DOGMA)^74^, and all tRNA genes were predicted using tRNAscan-SE v1.3.1^75^ and ARAGORN v1.2.38^76^. The plastome was deposited in GenBank (accession number MG873498). Circular and linear plastome maps were drawn with OGDRAW v1.2^77^. Secondary structures were predicted using the tRNAscan-SE 2.0 web server^78^.

Dispersed repeat sequences were identified by performing “blastn” searches using BLAST + v2.6.0^79^ against itself, with a word size of 11, an *e*-value of 1 × 10^−10^, and at least 90% sequence identity. Tandem repeat sequences in the plastomes were identified using Tandem Repeats Finder v4.09^80^ with default parameters. Two other Papaveraceae plastomes (*Papaver somniferum*; NC_029434 and *Coreanomecon hylomeconoide*; NC_031446) were examined for repeat sequences.

### Genome structural analyses

To confirm the structural divergence of the *Lamprocapnos* plastome, PCR was carried out using the total genomic DNA and primers designed with Primer3^81^ in Geneious R7 (Table S4). Each reaction was 25 μl in volume, including 19.375 μl of distilled water, 2.5 μl of 10 × *Taq* Reaction Buffer, 0.5 μl of 10 mM dNTPs, 0.125 μl of DiaStar^TM^ *Taq* polymerase (5 units/μl, Solgent Co., Daejeon, South Korea), 0.5 μl of each primer (10 pmole/μl), and 1 μl of total genomic DNA (20 ng). All reactions included an initial denaturation step (95 °C for 2 min), 35 cycles of denaturation (95 °C for 20 s), annealing (60 or 62 °C for 40 s), and extension (72 °C for 1 min 30 to 3 min 10 s, depending on the size of the target region) and final extension (72 °C for 5 min). Amplification products were evaluated by running on 1.5% agarose gels.

The *Lamprocapnos* plastome and the two published Papaveraceae plastomes, *P. somniferum* and *C. hylomeconoide*, were aligned with the outgroup *Euptelea pleiosperma* (Eupteleaceae, NC_029429) from Ranunculales using the “progressiveMauve” algorithm in Mauve v2.3.1^82^ in Geneious R7. To reconstruct the history of inversions and gene relocations in the *Lamprocapnos* plastome, gene order and orientation in the genome were compared with the inferred ancestral plastome architecture for angiosperms^1^ using Genome Rearrangements In Man and Mouse (GRIMM) v2.0.1^83^ with the numbered LCBs identified using Mauve. For these analyses, one copy of the IR was removed from the whole plastome to reduce the genomic complexity of the IR contraction and expansion changes.

To verify the single-nucleotide polymorphisms in three *trnI_CAU* copies, specific primer pairs were used (Table S5). The PCR products were purified using the Solg^TM^ Gel & PCR extraction system (Solgent Co., Daejeon, South Korea) following the manufacturer’s protocol.

Sequencing of PCR products was performed using an ABI 3730xl DNA Analyzer (Applied Biosystems, California, USA) at Solgent Co.

### RNA isolation and reverse transcription-PCR

Total RNA was isolated from the KNA fresh leaves using the methods of Ghawana *et al*.^84^ and treated with DNase I (Invitrogen, USA) to remove any trace of genomic DNA. To confirm whether the *accD* and *orf431* genes were transcribed, reverse transcription (RT)-PCR was performed using random hexamers and ImProm-II^TM^ Reverse Transcriptase (Promega, USA). PCR amplification was carried out with primer pairs specific to *accD* and *orf431* (Table S5). PCR purification, and sequencing were performed as described above.

### Survey of variability in the plastid *accD* and *ycf1* genes

Fresh leaves were obtained from five individuals of *L. spectabilis* from the Daegu Arboretum, Daegu, South Korea. The genomic DNA was extracted using the DNeasy® Plant Mini Kit (QIAGEN) following the manufacturer’s protocol. To test length variation in the *accD* and *ycf1* genes at the individual level, the variable regions were amplified via PCR using appropriate primers (Table S5). PCR amplification, purification, and sequencing were performed as described above. Amino acid repeats in the *accD* and *ycf1* genes were identified through rapid automatic detection and alignment of repeats in protein sequences (RADAR)^85^ and manually adjusted. Coiled-coil regions in proteins were predicted by COILS^86^ with the MTIDK matrix.

### Estimation of sequence divergence

Nonsynonymous and synonymous substitution rates were calculated in PAML v4.8^87^ using the CODEML program, employing the F3 x 4 codon frequency model, and gapped regions were excluded with the “cleandata = 1” option. All 79 protein-coding genes in the *Lamprocapnos spectabilis* plastome were selected for rate analysis. The sequenced plastomes from *P. somniferum*, *C. hylomeconoide* and *E. pleiosperma* were used. Individual gene alignments were generated based on the back-translation approach with MAFFT v7.017^88^ in Geneious R7. A constraint tree for all rate analyses was generated using maximum likelihood (ML) methods in RAxML v8.0.26^89^, employing the ‘GTRGAMMA’ model, with the rapid bootstrap algorithm (1,000 replicates). ML analysis was performed on a single alignment of the 79 protein-coding genes. Likelihood ratio tests (LRTs) were performed to test *d*_N_/*d*_S_ changes. A null model fixed across the entire tree, whereas an alternative model allowed different values of *d*_N_/*d*_S_ for branches in the phylogenetic tree. Statistical analyses were conducted with R v. 3.4.2^90^, and the Bonferroni correction for multiple comparisons was applied.

### Identification of intracellular gene transfer

To evaluate potential IGT, transcriptomes from *P. somniferum* were assembled *de novo* using the Sequence Read Archive (SRA) (ERR706833) with Trinity v2.2.0^91^. A nuclear-encoded *rps15* copy was identified in the transcriptome using BlastN (e-value cutoff of 1e-10), employing plastid-encoded *rps15* from *C. hylomeconoides* as the query sequence. The chloroplast transit peptide (cTP) and its cleavage site were predicted by TargetP v1.1^92^.

The *Lamprocapnos spectabilis* plastome and gene sequences are available on GenBank (MG873488-MG873498, MH319712-MH319716).

## Electronic supplementary material

Supplementary information
