# Supplementary material for: Reconfiguration of the plastid genome in Lamprocapnos spectabilis: IR boundary shifting, inversion, and intraspecific variation
Source: Sci Rep. 2018 Sep 11;8:13568. doi: 10.1038/s41598-018-31938-w (PMC6134119; doi:10.1038/s41598-018-31938-w)
Supplement: Supplementary file 1 — Supplementary information [file 41598_2018_31938_MOESM1_ESM.pdf]

Article title: Reconfiguration of the plastid genome in *Lamprocapnos spectabilis*: IR boundary shifting, inversion, and intraspecific variation

Authors: Seongjun Park, Boram An, and SeonJoo Park

Supplementary information

Figure S1. Plastome map for *Lamprocapnos*.

Figure S2. Duplication of the *trnI-CAU* gene in the *Lamprocapnos spectabilis* plastome.

Figure S3. Nucleotide sequence alignment of *Lamprocapnos accD* from complete and partial copies.

Figure S4. Amino acid sequence alignment of the plastid-encoded *accD* of *Lamprocapnos* with three copies from related species belonging to *Coreanomecon*, *Papaver*, and *Euptelea*.

Figure S5. Results of RT-PCR and transmembrane helix prediction.

Figure S6. Characterization of the coiled-coil domains within the plastid-encoded *accD* genes from *Lamprocapnos, Coreanomecon*, *Papaver*, and *Euptelea*.

Figure S7. Amino acid sequence alignment of the plastid-encoded *ycf1* of *Lamprocapnos* with three copies from related species belonging to *Coreanomecon*, *Papaver*, and *Euptelea*.

Figure S8. Characterization of the coiled-coil domains of the *ycf1* genes from *Lamprocapnos, Coreanomecon*, *Papaver*, and *Euptelea*.

Figure S9. Correlation of the nonsynonymous and synonymous substitution rates of the plastid-encoded genes.

Figure S10. Nonsynonymous and synonymous divergence was plotted against genomic positions. The vertical lines indicate the 14 breakpoints predicted by Mauve.

Figure S11. Gene transfer of plastid-encoded *rps15*.

Figure S12. Assay results using primers designed to amplify 11 regions.

Table S1. Comparison of the organization of Papaveraceae plastomes.

Table S2. NCBI accession numbers for length variation in the plastid *accD* and *ycf1* genes.

Table S3. Log likelihood scores used in likelihood ratio tests to test the fit of model H_1 (d_N/d_S values not constrained in the branch leading to *Lamprocapnos*) to H_0 (universal d_N/d_S values across entire tree).

Table S4. Primers used for confirming rearrangements.

Table S5. Primers used to test plastid-encoded *accD*, *ycf1*, *orf431* and three *trnI-CAU* genes.

Figure S1. Plastome map for *Lamprocapnos*. Dark green lines on the outside circle indicate the inverted repeats. Genes on the inside and outside of each map are transcribed in clockwise and counterclockwise directions, respectively. Tandem repeats are shown in red. Coverage is plotted on the inner circle. Blue lines within the inner circle indicate the positions of the pairs of repeats, with crossed connecting lines denoting reverse repeats.

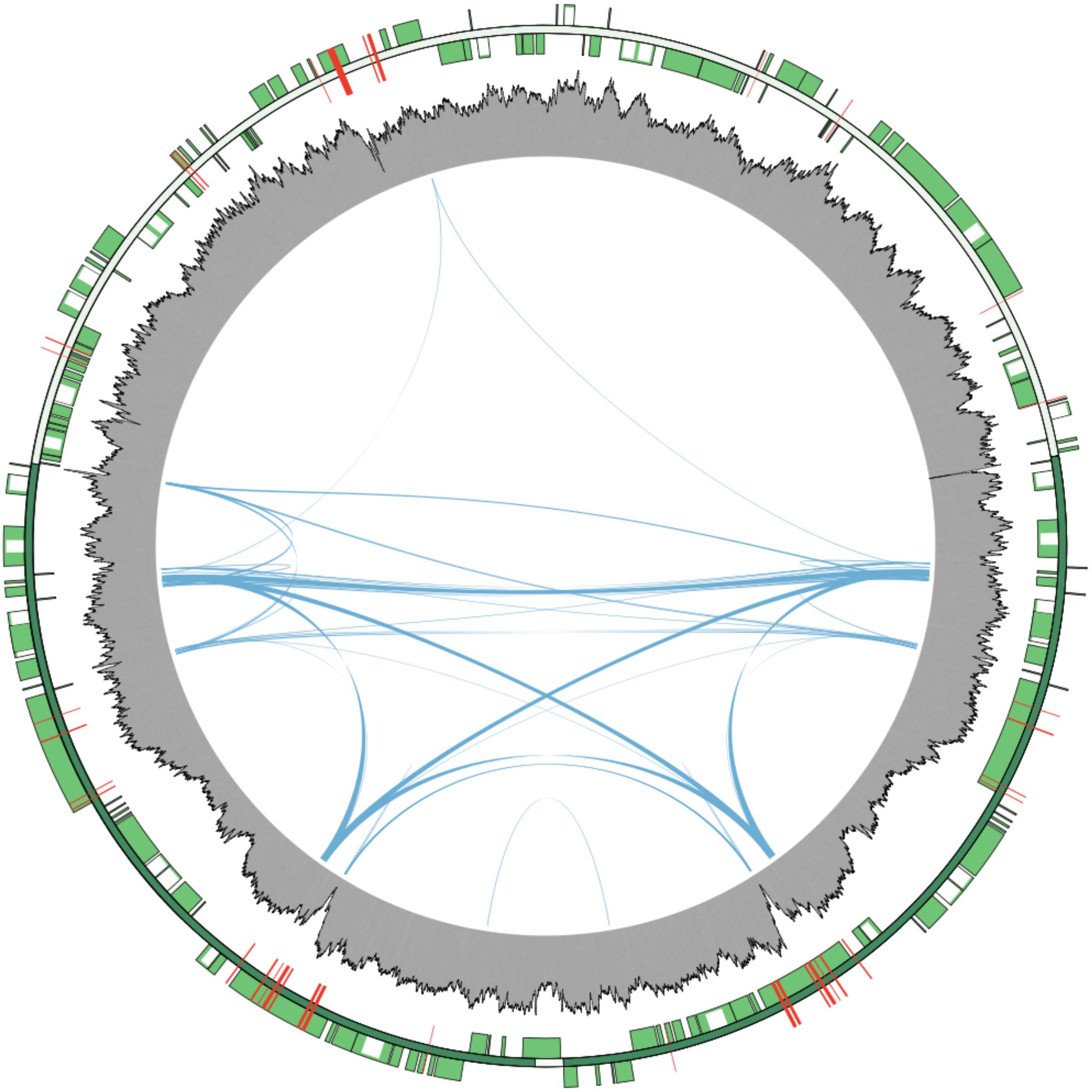

Figure S2. Duplication of the *trnI-CAU* gene in the *Lamprocapnos spectabilis* plastome. (A) Schematic diagram of the genomic regions surrounding the three *trnI-CAU* copies. Thick lines represent the IR, and thin lines represent the adjacent large single-copy (LSC) region. The numbers “1”, “2”, and “3” represent paralogs of *trnI-CAU* (red), and ϕ denotes a pseudogene. (B) Nucleotide alignment of *Lamprocapnos trnI-CAU* copies with three copies from related species belonging to *Coreanomecon*, *Papaver*, and *Euptelea*. The sequence logo indicates the most conserved bases at each position. The numbers correspond to each paralog of *trnI-CAU* in (A). (C) Predicted secondary structures for the three *trnI-CAU* sequences of *Lamprocapnos*. The substitution sites are highlighted with red circles. Asterisks indicate the predicted change in the D- and T-stems of secondary structures resulting from nucleotide substitutions.

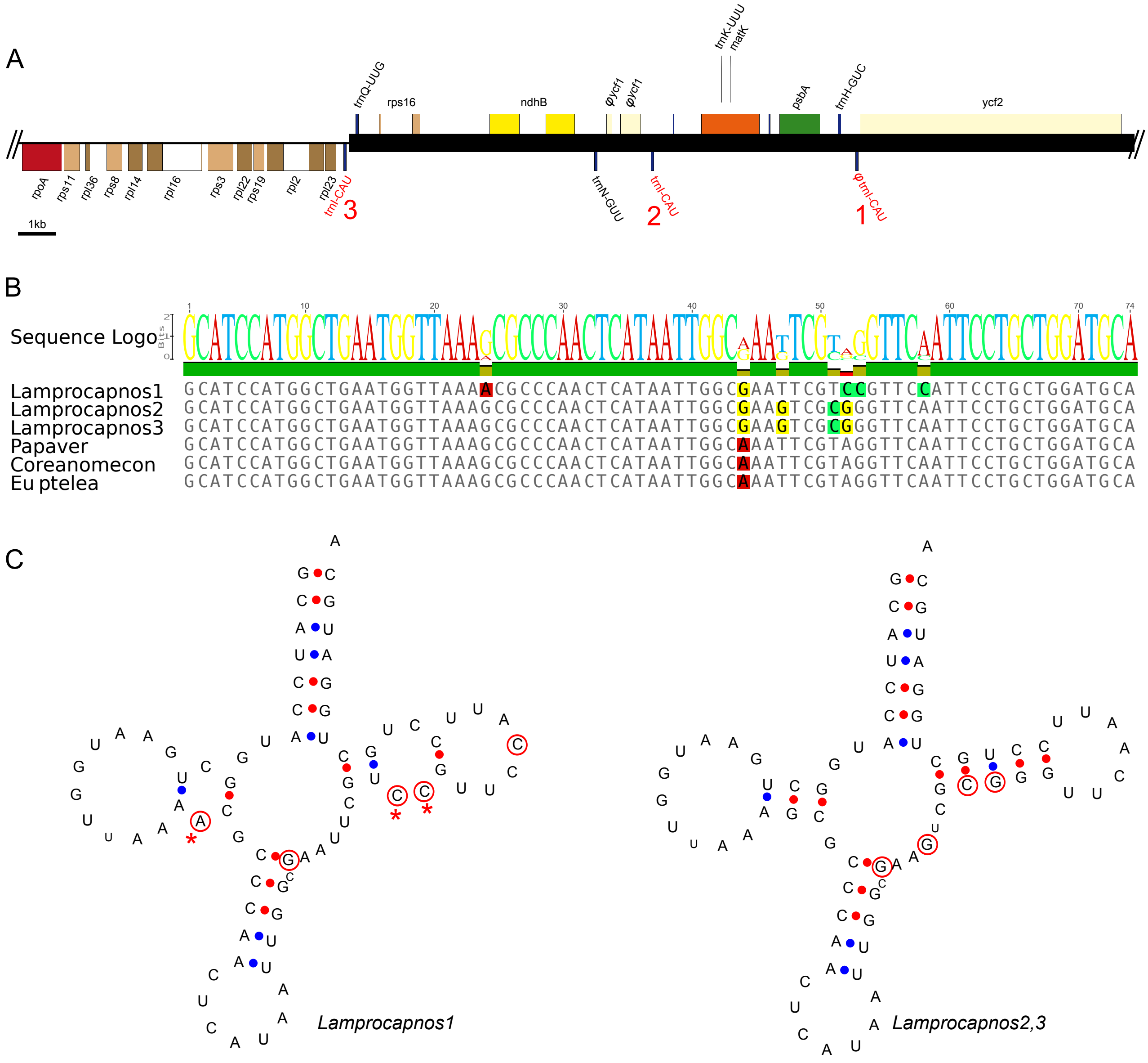

Figure S3. Nucleotide sequence alignment of *Lamprocapnos accD* from complete and partial copies.

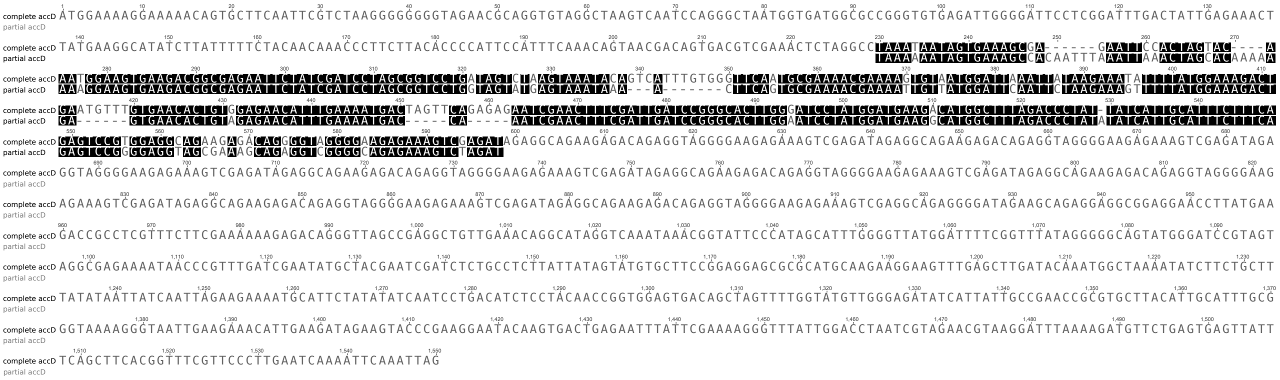

Figure S4. Amino acid sequence alignment of the plastid-encoded *accD* of *Lamprocapnos* with three copies from related species belonging to *Coreanomecon*, *Papaver*, and *Euptelea*.

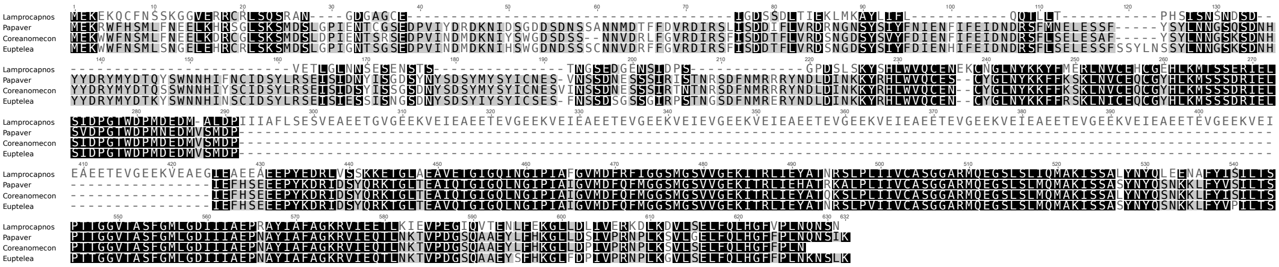

Figure S5. Results of RT-PCR and transmembrane helix prediction. (A) Lane M: SolGent^{TM} 1 kb plus DNA ladder, lane 1: plastid *rbcL*, lane 2: *orf431*, lane 3: plastid *accD*, lane 4: *orf431* + the *accD*, lane 5: plastid *clpP*, lane 6: negative control. For the plastid *clpP* gene, we designed a PCR screen that specifically targets the intron-containing version, which is expected to be 1,968 bp in size, as an alternative control and mRNA of *clpP* product must be 627 bp in size. If the RNA sample has genomic DNA contamination, two different sizes of results will be obtained. (B) Prediction of transmembrane helices for *orf431*. Red lines indicate transmembrane regions; blue lines indicate intracellular regions; and pink lines indicate extracellular regions. The vertical axes indicate the probability of transmembrane helices, and the horizontal axes indicate amino acid sequences.

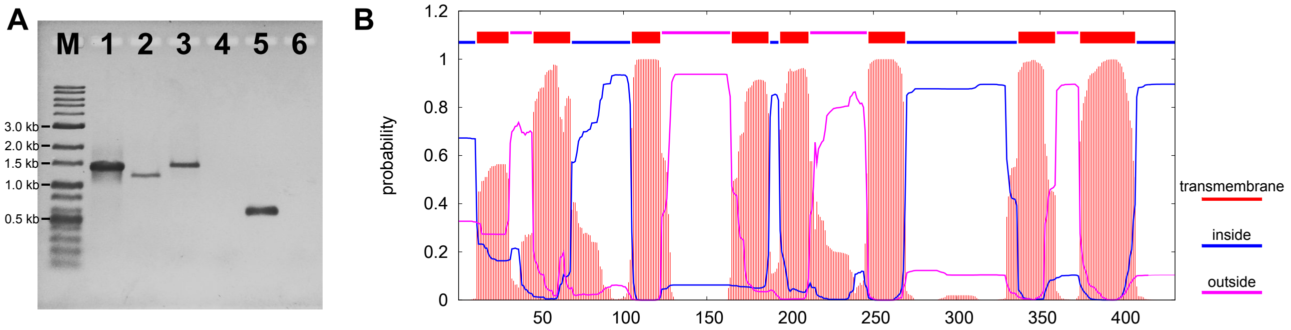

Figure S6. **Characterization of the coiled-coil domains within the plastid-encoded *accD* genes from *Lamprocapnos, Coreanomecon*, *Papaver*, and *Euptelea*.** COILS predicted coiled-coil regions between the two conserved domains, with a high probability of coiled-coil formation obtained in scanning windows of 14,21, and 28 residues.

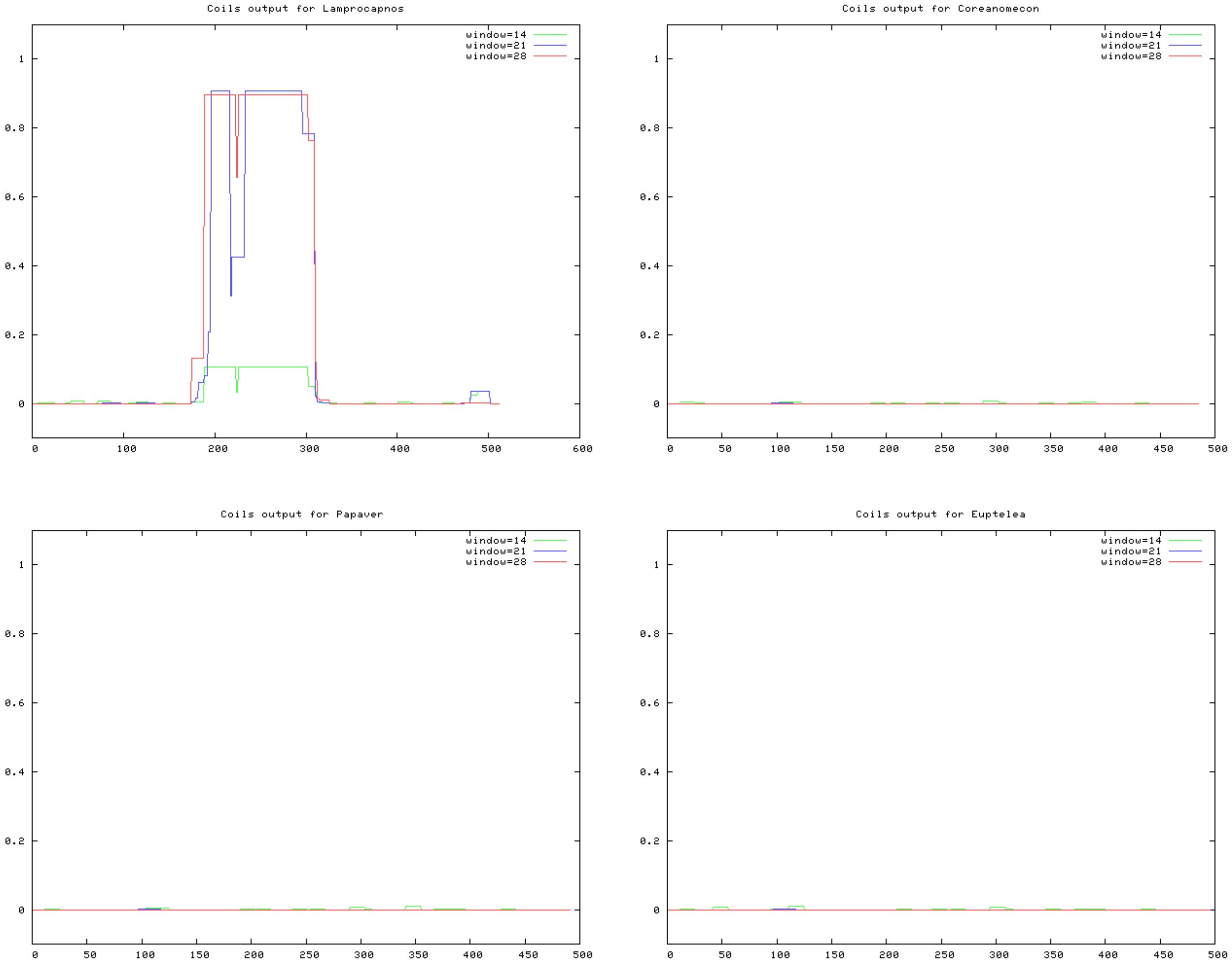

Figure S7. Amino acid sequence alignment of the plastid-encoded *ycf1* of *Lamprocapnos* with three copies from related species belonging to *Coreanomecon*, *Papaver*, and *Euptelea*.

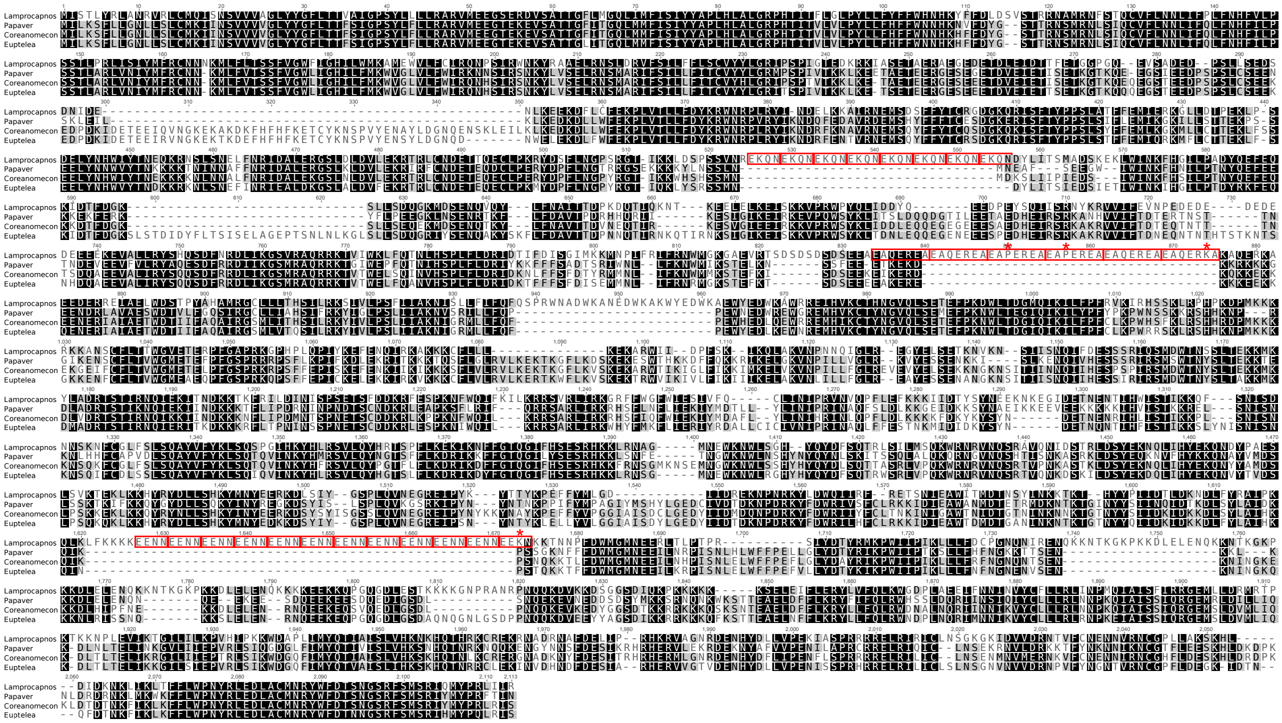

Figure S8. Characterization of the coiled-coil domains of the *ycf1* genes from *Lamprocapnos, Coreanomecon*, *Papaver*, and *Euptelea*. COILS predicted coiled-coil regions between the conserved domains, with a high probability of coiled-coil formation obtained in scanning windows of 14,21, and 28 residues.

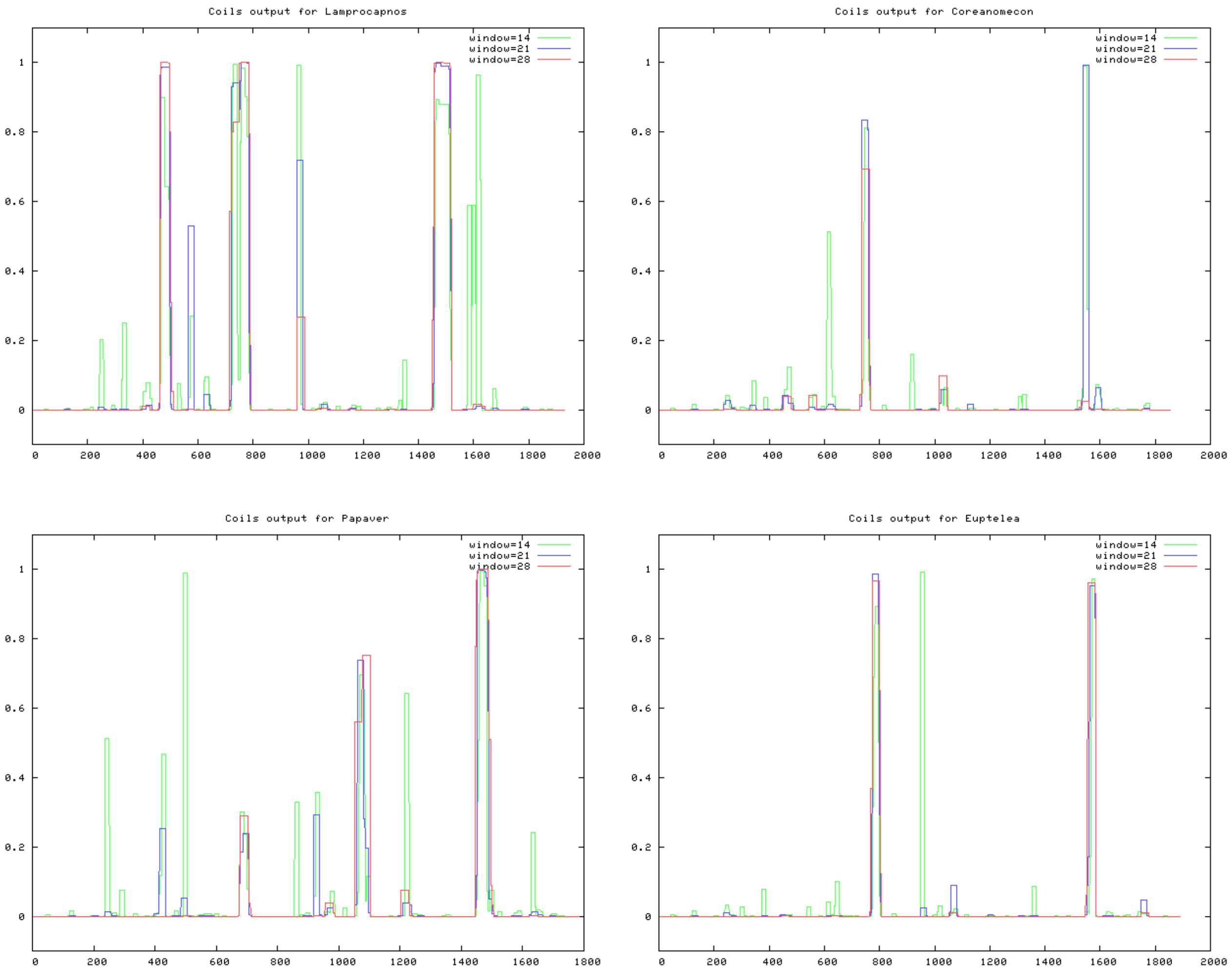

Figure S9. Correlation of the nonsynonymous and synonymous substitution rates of the plastid-encoded genes.

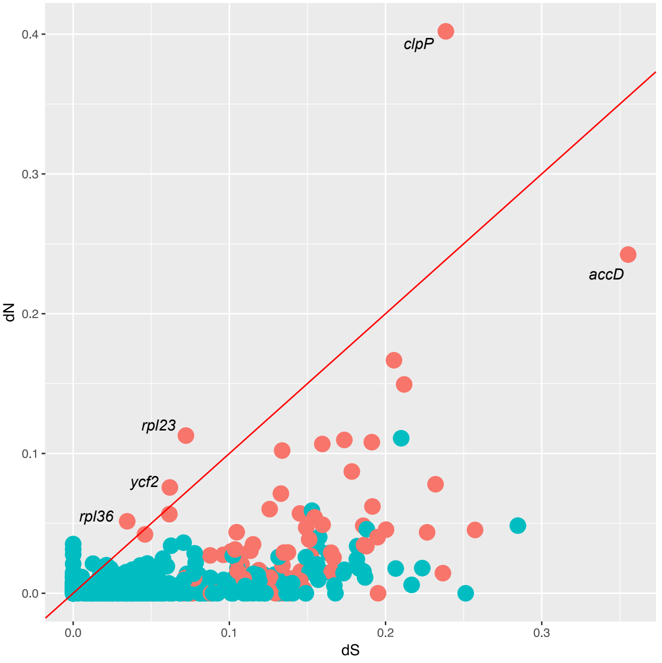

Figure S10. Nonsynonymous and synonymous divergence was plotted against genomic positions. The vertical lines indicate the 14 breakpoints predicted by Mauve.

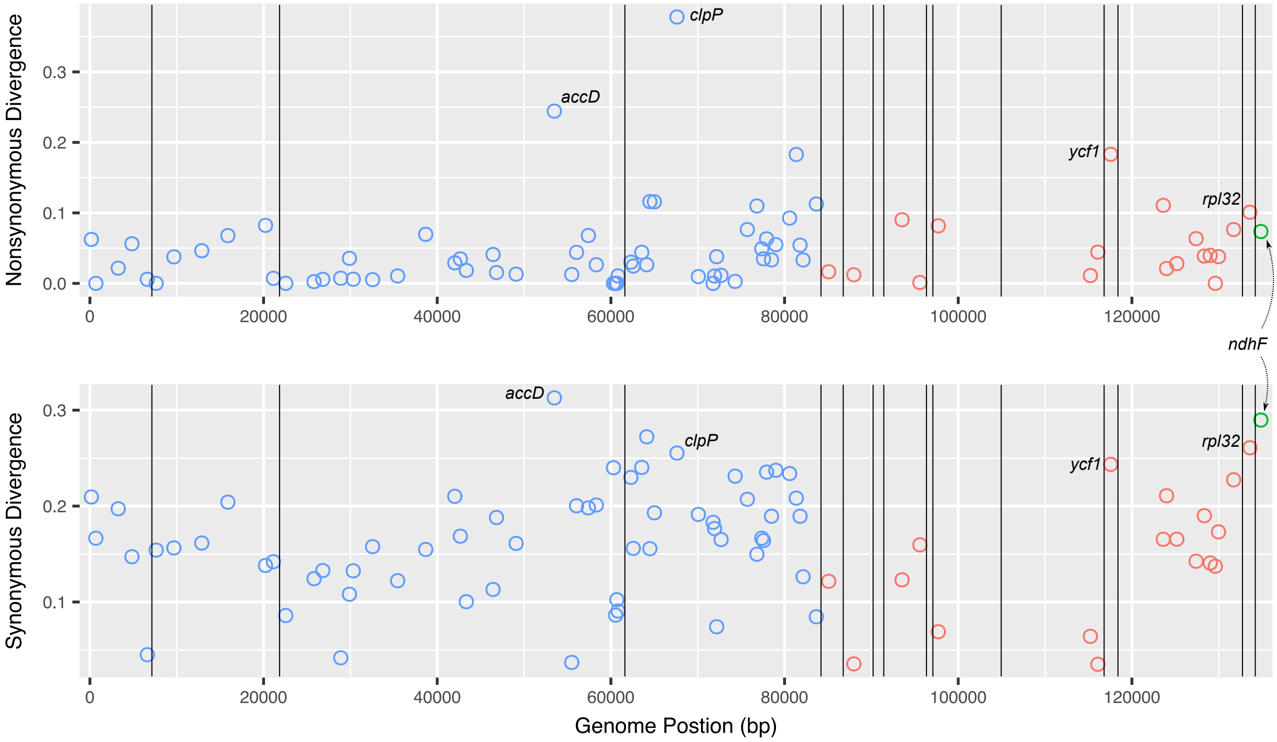

Figure S11. Gene transfer of plastid-encoded *rps15*. (A) Nucleotide and amino acid sequence alignment of the *rps15* of *Lamprocapnos*, *Coreanomecon*, *Papaver*, and *Euptelea*. The black box indicates the *rps15* gene region annotated in a previous study[27]. The red box indicates a conserved domain of ribosomal protein S15. (B) Nucleotide and amino acid sequences of the nuclear-encoded *rps15* gene from *Papaver*. Green boxes indicate plastid transit peptides (TP) predicted using TargetP. The red box indicates a conserved domain of ribosomal protein S15.

A

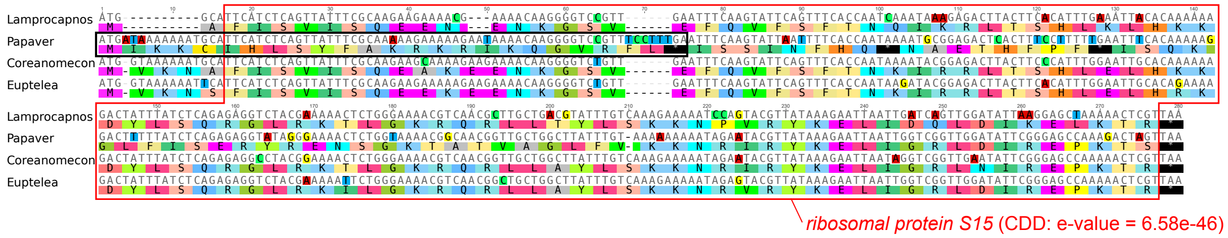

B

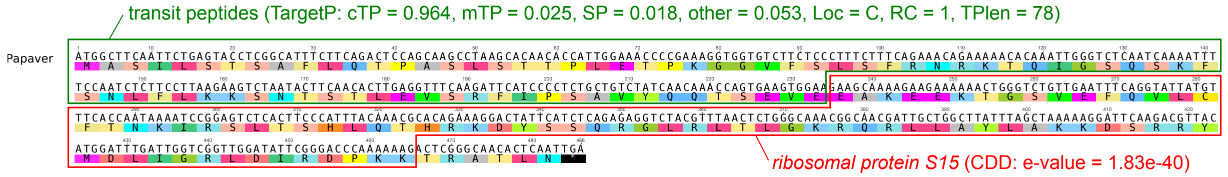

Figure S12. Assay results using primers designed to amplify 11 regions. Lane M contains the SolGent^{TM} 1 kb plus DNA ladder, and lanes (1-11) correspond to the numbers of the 11 PCR amplicons on Figure 3A or 3C.

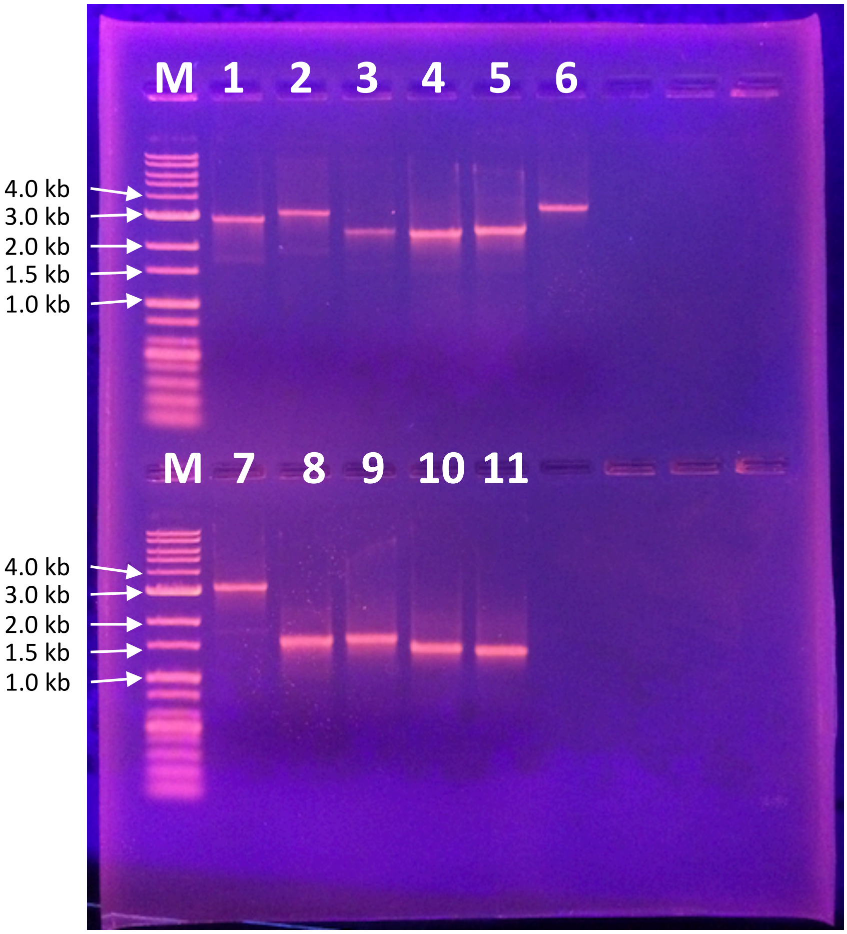

Table S1. Comparison of Papaveraceae plastome organization.

	Lamprocapnos spectabilis	*Papaver somniferum*	*Coreanomecon hylomeconoide*
Genome size (bp)	188,754	152,931	158,824
LSC length (bp)	83,341	83,029	86,916
SSC length (bp)	1,645	17,920	18,538
IR length (bp)	51,384	25,991	26,685
Number of different genes	113	112	113
protein genes (duplicated in IR)	79 (18)	78* (6)	79 (6)
tRNA genes (duplicated in IR)	30 (11)	30 (7)	30 (7)
rRNA genes (duplicated in IR)	4 (4)	4 (4)	4 (4)
introns	21 (7)	21 (5)	21 (5)
Dispersed repeats (bp)	4672	180	0
Tandem repeats (bp)	3085	1037	1145
GC content (%)	39.2	38.9	38.7

*Although *rps15* of *Papaver somniferum* is annotated in GenBank as an intact gene, new analysis suggests it is a pseudogene.

Table S2. NCBI accession numbers for length variation in the plastid *accD* and *ycf1* genes. The numbers "1", "2", and "3" represent hotspot regions of the *ycf1* gene.

Taxon	Abbreviation	*accD*	*ycf1* (1 & 2)	*ycf1* (3)
Lamprocapnos spectabilis	DA1	MG873493	MG873488	MH319712
	DA2	MG873494	MG873489	MH319713
	DA3	MG873495	MG873490	MH319714
	DA4	MG873496	MG873491	MH319715
	DA5	MG873497	MG873492	MH319716

Table S3. Log likelihood scores used in likelihood ratio tests to test the fit of model H_1 (d_N/d_S values not constrained in the branch leading to *Lamprocapnos*) to H_0 (universal d_N/d_S values across entire tree). Bold font indicates a significant correlation with $p < 0.05$.

Gene	d_N/d_S	lnL H_0	lnL H_1	$2*(H_1-H_0)$	*p*-value	d.f.	Bonferroni
clpP	1.6851	-1573.931947	-1562.883224	22.097446	2.59E-06	1	**0.000013**
rpl23	1.5616	-498.330765	-495.151309	6.358912	0.01167931	1	0.05839655
rpl36	1.4913	-203.935608	-197.536676	12.797864	0.000347015	1	**0.001735077**
ycf2	1.2231	-12403.26176	-12391.28393	23.955656	9.86E-07	1	**0.00000493**

Table S4. Primers used for confirming rearrangements.

PCR set	Forward	Sequence (5'->3')	Reverse	Sequence (5'->3')	Expected size (bp)
1	22632F	TGCTACTGCACTGTTCATTTTAGT	19685R	TATCCGAAGCGAGTTTTCAAGAGA	2948
2	9959F	CTTGCCTATTCTTACCGGTTTTCC	6701R	AGTACCTCGTATTTTACCCTCTGC	3259
3	849F	TATTTTATTCCTCACGCCCAGGAT	86049R	GATCTCTTCCTTCTCTTCGGGATC	2557
4	83582F	ATTCTTTCGTGGATTGGGTTTCAC	86049R	GATCTCTTCCTTCTCTTCGGGATC	2468
5	86026F	GATCCCGAAGAGAAGGAAGAGATC	88568R	TTTCGTAGTAGCCTCATTAGACCG	2543
6	90606F	AAGGTATTGACGGGGATTCTCAAA	94044R	GAATGGGGTGGGGTATTAGCATAT	3439
7	96125F	GAAGCTTTTCTGATGGTATGCCTC	99312R	CCTGGAAGGGACAAAAGAAACATC	3188
8	104492F	GGCTGTTGGATCAAATGACAAAGA	106108R	TCAAGAATTAGGGCCTCACAATCA	1617
9	116489F	AATTAGAGGCTGCTAACAAAGGGA	118142R	TGACCAATGAACCAACCAACAAAA	1654
10	134035F	CAACACCATTCGTAATTCCACCAA	136418R	ACTCTGTCTGCTGCCCTATTATTC	1426
11	136438F	AATAATAGGGCAGCAGACAGAGTC	134035F	CAACACCATTCGTAATTCCACCAA	1474

Table S5. Primers used to test plastid-encoded *accD*, *ycf1*, *orf431* and three *trnI-CAU* genes. The numbers "1", "2", and "3" after each *ycf1* primer represent hotspot regions of the *ycf1* gene. The numbers "1", "2", and "3" after each *trnI* primer represent paralogs correspond to the numbers of *trnI-CAU* in supplementary Figure 2.

PCR set	Forward	Sequence (5'->3')	Reverse	Sequence (5'->3')
RT-PCR	rbcLF	GTCACCACAAACAGAGACTAAAGC	rbcLR	TGATCTCCTTCCATACCTCACAAG
	orf431F	AACACCCAAATCACATCGGTAATC	orf431R	TGCCTAAGAAAAGAGCTTTCCGTA
	accDF	TGGAAAAGGAAAAACAGTGCTTCA	accDF	CCTTACGTTCTACGATTAGGTCCA
	clpPF	ATTCCAAAAGTAGCCTTTCGCATT	clpPR	TTCATGGAGTCTCAAGATTCTCGC
ycf1	ycf1_1&2F	CTAGAAAGGGGGTCTCTTGATCTG	ycf1_1&2R	CGTATGGAGTACTATCCCACAGTT
	ycf1_3F	ATATTGAGGCCTGGATCACTATGG	ycf1_3R	TAGGTTTTTGAATGTCTGATCCGC
trnI-CAU	trnI_1F	CTTGTGCATTAGGAAACCAGCTAC	trnI_1R	ATGTGAGTGAAAGATCCCATGGAA
	trnI_2F	AAACCAGCTACCCATTCGTTATCT	trnI_2R	TCGATTGATCCACGATCTAATTCT
	trnI_3F	TCTCCAACCATAACCAAACACTTT	trnI_3R	CAATTCCCGCCCAAATTTCTATGA
